# The impact of pharmacological thromboprophylaxis and disease-stage on postoperative bleeding following colorectal cancer surgery

**DOI:** 10.1186/s12957-019-1653-1

**Published:** 2019-06-27

**Authors:** Hiroyuki Ohta, Toru Miyake, Tomoharu Shimizu, Hiromichi Sonoda, Tomoyuki Ueki, Sachiko Kaida, Tsuyoshi Yamaguchi, Hiroya Iida, Masaji Tani

**Affiliations:** Department of Surgery, Shiga University of Medical Science, Seta-tsukinowacho, Otsu, Shiga 520-2192 Japan

**Keywords:** Bleeding, Complications, Antithrombotic prophylaxis, Fondaparinux, Anoxaparin, D-dimer

## Abstract

**Background:**

Pharmacological thromboprophylaxis after colorectal cancer (CRC) surgery is internationally recommended for venous thromboembolism (VTE) prevention. The aim of this retrospective study was to evaluate the risk factors of postoperative bleeding after elective surgery for patients with primary CRC receiving pharmacological thromboprophylaxis of fondaparinux or enoxaparin.

**Methods:**

We experienced consecutive 266 patients who underwent elective surgery for CRC during the study period. Finally, the medical records of 218 patients with CRC administrated fondaparinux or enoxaparin following surgery were retrospectively reviewed to evaluate symptomatic VTE until 28 days and postoperative bleeding comparing perioperative D-dimer levels.

**Results:**

The significant differences in TNM classification staging and type of thromboprophylaxis were observed between postoperative bleeding-negative and bleeding-positive group. There was no statistical significance among other backgrounds of patients between the two groups. One case (0.46%) of symptomatic VTE and total 11 cases (5%) of postoperative bleeding were observed. In the univariate analysis, fondaparinux thromboprophylaxis and early disease-stage CRC (stages 0 and I) were associated with risk for postoperative bleeding. Multivariate analysis revealed that fondaparinux thromboprophylaxis was identified as an independent risk factor of postoperative bleeding. Moreover, preoperative levels of D-dimer in patients with stage IV CRC were significantly higher than those with the other stages. The significant elevation in preoperative D-dimer was also observed in patients with stage II CRC compared to those with stage I CRC. Perioperative levels of D-dimer in patients with advanced disease-stage CRC (stages II, III, and IV) were significantly higher than those in patients with early disease-stage CRC.

**Conclusions:**

Fondaparinux administration and early disease-stage CRC appeared to be risk factors for postoperative bleeding in patients with pharmacological thromboprophylaxis undergoing surgical treatment for CRC. Patients’ hypercoagulative condition depending on disease progression of CRC might be related to the occurrence of postoperative bleeding following CRC surgery.

## Background

Since venous thromboembolism (VTE) can result in fatal outcomes due to pulmonary thromboembolism, it is important to prevent VTE for patients undergoing abdominal surgery [1]. Recent guidelines recommended using physiological therapies such as intermittent pneumatic compression (IPC) and elastic stockings on the lower legs or pharmacological thromboprophylaxis according to patient risk level following abdominal major surgery [2]. There is a growing body of evidence supporting a symbiotic relationship between the clotting system and the biology of cancer. Colorectal cancer (CRC) leads to increased activation of the clotting system which manifests VTE; therefore, pharmacological thromboprophylaxis after CRC surgery is internationally recommended for VTE prevention [3, 4]. Two anticoagulants, fondaparinux (a synthetic Xa inhibitor) [5] and enoxaparin (a low molecular weight heparin) [6–9], are available for use in the clinical setting for such patients in Japan. A recent systematic review demonstrated that there was no difference between perioperative thromboprophylaxis with enoxaparin compared with fondaparinux in their effects on mortality, thromboembolic outcomes, major bleeding, or minor bleeding in people with cancer [10]; however, the risk factors associated with postoperative bleeding in patients with pharmacological thromboprophylaxis following CRC surgery remain unknown. Therefore, we retrospectively evaluated the risk factors of postoperative bleeding after elective surgery for patients with primary CRC receiving perioperative pharmacological thromboprophylaxis comparing perioperative D-dimer levels.

## Methods

### Patients

We experienced consecutive 266 patients who received elective surgery for treatment of primary CRC from June 2010 to December 2013 at the Shiga University of Medical Science Hospital. Patients received recommended VTE preventive treatments according to their classification of risk by type of surgery, found in the Japanese Circulation Society (2009) guidelines [2]. Patients with a greater than high risk received IPC and pharmacological thromboprophylaxis. Thirty-six patients who received unfractionated heparin prior to surgery due to arrhythmia and cardiac and cerebral vascular lesions were excluded in this study. Nine patients with high risk and three patients with a lesser than intermediate risk who received only IPC without anticoagulants were also excluded in this study. Finally, 218 patients were included in this retrospective analysis. Out of 218 patients, 151 patients received enoxaparin as thromboprophylaxis at a dose of 2000 IU × 2/day from August 2010 to May 2012 and from May 2013 to December 2013, and 67 patients were also administered fondaparinux at a dose of 1.5 or 2.5 mg/day according to their renal function from June 2012 to April 2013. The epidural anesthesia catheter was removed at the second postoperative day (POD2). After a minimum of 2 h post-removal of the epidural catheter, the anticoagulants were administrated until POD7. The medical records were retrospectively reviewed to evaluate symptomatic VTE and postoperative bleeding. Patients’ clinicopathological parameters and routine laboratory data (serum creatinine, alanine aminotransferase, total-bilirubin, carcinoembryonic antigen, carbohydrate antigen 19-9, and D-dimer) were also retrospectively collected. This study has been performed in accordance with the ethical standards as laid down in the 1964 Declaration of Helsinki. This study was approved by the ethical committee of Shiga University of Medical Science (approved IRB number R2017-021).

### Evaluation of postoperative bleeding and VTE

The evaluation of postoperative bleeding was divided into two categories as described previously [11, 12]. Briefly, a major bleeding was classified as an event which met at least one of the following definitions: fatal bleeding, retroperitoneal or intracranial bleeding, bleeding of critical organs (intraocular, adrenal, endocardial, intestinal or spinal bleeding), surgical site bleeding that required surgical intervention, clinically overt bleeding with a decrease in hemoglobin by at least 20 g/L, or the need for a transfusion of two units of red blood cells or whole blood. Minor bleeding was defined as a hemorrhage that did not meet any of the major bleeding criteria.

The symptomatic VTE were reviewed until POD28 to evaluate the efficacy of VTE preventive treatments. Whole body-enhanced CT scan of the neck to the lower legs was performed to rule out VTE when the symptoms (dyspnea, chest pain, edema, pain in lower legs, etc.) of VTE were observed. The adverse events and postoperative complications were evaluated according to Common Terminology Criteria for Adverse Events version 4.0 (CTCAE ver.4) and Clavien-Dindo classification [13].

### Statistical analysis

The continuous variables were expressed as median (25th–75th quartile) and analyzed with the Wilcoxon signed-rank test. The categorized variables were analyzed with Fisher’s exact chi-squared tests. Univariate and multivariate logistic regression analysis was used to identify independent risk factors for postoperative bleeding. We followed standard methods to estimate sample size for multivariate logistic regression, with at least ten outcomes needed for each included independent variable [14, 15]. An expected bleeding event rate was 10%, which was calculated from data in the previous studies that evaluated bleeding events in patients administrated fondaparinux and enoxaparin after CRC surgery [16–21]. We estimated to require 200 attempts (20 bleeding events) to appropriately perform multiple logistic regression with two variables. A *p* value of < 0.05 was considered to indicate statistical significance. Statistical analyses were carried out using JMP 9.0.2 (SAS Institute Inc., NC, USA)

## Results

### Patients’ background

Fondaparinux prophylaxis was often used compared to enoxaparin in the postoperative bleeding-positive group; therefore, we included the type of pharmacological thromboprophylaxis agents in the univariate and multivariate analysis. We also observed a statistically significant difference in TNM classification stage between two groups (Table 1). There was no statistical significance among backgrounds of patients, preoperative laboratory examinations, and the incidence of preoperative comorbidities between postoperative bleeding-negative and bleeding-positive groups. No statistical significance was observed in surgical approach (open and laparoscopic), blood loss, duration of surgery (open and laparoscopic), use of epidural anesthesia, and tumor location (right colon, left colon, and rectum) between the two groups (Table 1).

**Table 1:** Clinical characteristics of the patients

	Postoperative bleeding (−), *N* = 207	Postoperative bleeding (+), *N* = 11	*p* value
Backgrounds of patients			
Age (years)	69 (61–74)	65 (60–74)	0.6270
Gender (male:female)	122:85	8:3	0.3524
Height (cm)	159.8 (152.7–166.6)	166.0 (154.1–170.8)	0.3006
Weight (kg)	54.7 (49.7–62.8)	64.0 (51.9–70.3)	0.0958
ASA-PS classification (1:2:3)	80:122:5	4:6:1	0.5673
Preoperative laboratory examinations			
Platelet (10^4^/mm^3^)	23.3 (18.3–26.9)	25.7 (21.5–28.6)	0.1377
eGFR	73.3 (61.3–85.8)	69.3 (64.2–78.3)	0.8283
Serum ALT (IU/L)	17 (11–24)	20 (14–26)	0.2967
Serum T-Bil (IU/L)	0.62 (0.50–0.92)	0.74 (0.46–0.77)	0.6609
D-dimer (μ/mL)	0.55 (0.3–1.1)	0.4 (0.3–0.7)	0.5690
CEA (ng/mL)	5.3 (2.8–11.8)	4.8 (2.5–11.3)	0.8202
CA19-9 (U/mL)	14 (8–23)	21 (10–40)	0.3140
Preoperative comorbidities			
Number of comorbidities (4:3:2:1:0)	1:3:15:59:129	0:1:0:3:7	0.4854
Hypertension	162	4	
Diabetes mellitus	29	2	
Cerebrovascular disease	7	1	
Ischemic heart disease	2	0	
Respiratory disease	9	0	
Surgical and tumor characteristics			
Approach (open:laparoscopic)	80:127	2:9	0.1505
Blood loss (g)	162 (50–403)	170 (50–229)	0.8022
Duration of surgery			
Open (min)	223 (181–408)	379 (162–597)	0.7841
Laparoscopic (min)	284 (227–342)	237 (216–313)	0.3000
Use of epidural anesthesia, *n* (%)	189 (91.3%)	10 (90.9%)	0.9641
Type of anticoagulants (fondaparinux:enoxaparin)	60:147	7:4	0.0374
Tumor location (Rt colon:Lt colon:Rectum)	58:57:92	4:2:5	0.8280
TNM classification (stage 0:I:II:III:IV)	11:51:52:58:35	1:6:1:0:3	0.0267

Data was presented as median (25th–75th percentile) and number (%). *BMI* body mass index, *ASA-PS* American Society of Anesthesiologists—physical status, *eGFR* estimated glomerular filtration rate, *ALT* alanine aminotransferase, *T-Bil* total-bilirubin, *CEA* carcinoembryonic antigen, *CA19-9* carbohydrate antigen 19-9, *TNM classification* UICC classification ver.7 Staging for adenocarcinoma

### Postoperative VTE and bleeding

Total 11 cases (5%) of postoperative bleeding, one case (0.46%) of major bleeding and ten cases (4.6%) of minor bleeding, were observed. The details of minor bleeding were four cases of intestinal bleeding, three cases of subcutaneous hematoma, and three cases of intra-abdominal bleeding. The discontinuation of anticoagulants due to postoperative bleeding was required in nine patients (4.1%). The univariate analysis revealed that early disease stage of CRC (stages 0 and I) and fondaparinux prophylaxis were associated with risk for postoperative bleeding in our study cohort. Multivariate analysis demonstrated that fondaparinux prophylaxis was identified as an independent risk factor for postoperative bleeding (Table 2). There were no cases of fatal bleeding during the study period. One case (0.46%) of symptomatic VTE was observed in a patient with the enoxaparin prophylaxis during the observational period.

**Table 2:** Univariate and multivariate analysis for risk factors of postoperative bleeding

		Univariate analysis		Multivariate analysis	
		OR (95% CI)	*p* value	OR (95% CI)	*p* value
Platelet	≤ 19.8 × 10^4^/mm^3^	1 (reference)			
	> 19.8 × 10^4^/mm^3^	4.892 (0.909–90.67)	0.0674		
Blood loss	≤ 290 g	1 (reference)			
	> 290 g	0.209 (0.011–1.225)	0.0720		
Approach	Laparoscopic	1 (reference)			
	Open	0.352 (0.053–1.412)	0.1505		
TNM classification	Stages II, III, and IV	1 (reference)		1 (reference)	
	Stages 0 and I	4.092 (1.192–16.09)	0.0252	2.683 (0.737–11.00)	0.1345
Type of anticoagulants	Enoxaparin	1 (reference)		1 (reference)	
	Fondaparinux	4.287 (1.248–16.87)	0.0209	4.864 (1.174–17.25)	0.0310

### Alteration in D-dimer

**Fig. 1:**
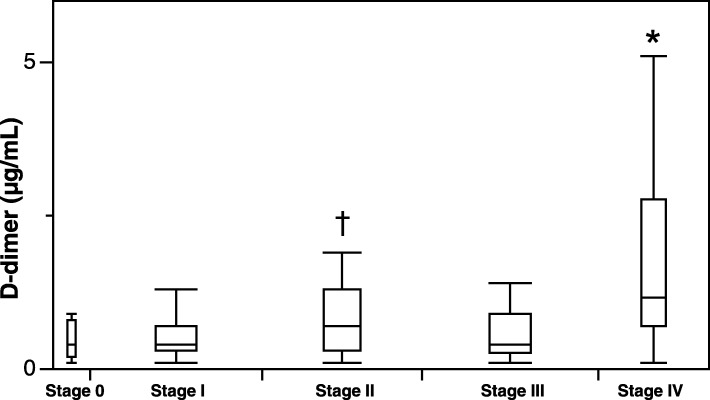
Preoperative levels of D-dimer according to the pathological TNM classification of CRC. *p < 0.05 vs. stages 0, I, II, and III. ^†^*p* < 0.05 vs. stages I

Preoperative levels of D-dimer in patients with stage IV CRC were significantly higher than those in patients with the other stages. The significant elevation in preoperative D-dimer was also observed in patients with stage II CRC compared to those in patients with stage I CRC (Fig. 1).

**Fig. 2:**
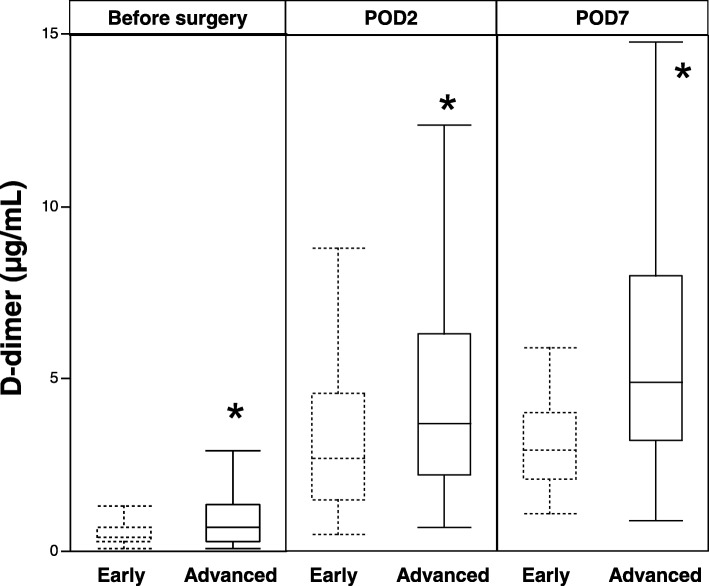
Perioperative levels of D-dimer according to disease stage of CRC. Early, early disease-stage CRC; advanced, advanced disease-stage CRC. *p < 0.05 vs. early disease-stage CRC

The levels of D-dimer before surgery, at second day, and at seventh day after surgery in patients with advanced disease stage of CRC (stages II, III, and IV) were significantly higher than those in patients with early disease stage of CRC (stages 0 and I) (Fig. 2).

## Discussion

The importance of VTE prevention is widely recognized in clinical medicine, since VTE can result in severe outcomes such as pulmonary embolism. In addition, the incidence of postoperative VTE following abdominal surgery in Japan has been increasing in recent years due to the change in lifestyle to a more sedentary Western lifestyle and improvements in the diagnostic accuracy of VTE [22, 23]. Both fondaparinux and enoxaparin demonstrate efficacy and safety for the prevention of VTE after abdominal surgery for malignancies [24]. A recent systematic review concluded that the risk of VTE in Asian general surgery patients is likely to be low [25]. These studies included patients with and without in-hospital anticoagulant administration. Kim et al. reported that the incidence of VTE was 3.7% (31/840 patients) in patients undergoing colorectal cancer surgery without perioperative anticoagulant prophylaxis [26]. The incidence of symptomatic VTE in patients with enoxaparin prophylaxis reportedly ranged from 0.43 to 1.4% [13, 27, 28] and 0 to 23.3% in patients with fondaparinux prophylaxis [16, 21, 29, 30] following abdominal surgery. One case (0.46%) of VTE was observed during our observational period. Thus, our findings were almost similar to the incidences of symptomatic VTE found in previous studies. However, the number of patients enrolled in this study seemed to be too small to compare the efficacy of the two anticoagulants prophylaxis in this study.

The latest meta-analysis demonstrated that fondaparinux was significantly more effective in preventing VTE after total hip replacement therapy when compared to enoxaparin, although this effectiveness was accompanied with an increased risk of major postoperative bleeding [31]. Only a few studies have compared the efficacy and safety between fondaparinux and enoxaparin in the same study cohorts following abdominal surgery. In those studies, postoperative bleeding events were reported as 21.2 % in the enoxaparin group and 37.6% in the fondaparinux group after major hepatobiliary-pancreatic surgery in Japanese patients [32]. Total postoperative bleeding was also observed at 11.5 % in the enoxaparin group and 22.2% in the fondaparinux group among the adult cancer population [19]. The postoperative transfusions for major postoperative bleeding increased by 0.9% in the fondaparinux group compared to 0.2% in the enoxaparin group following thoracic surgery [27]. According to previous comparison studies, postoperative bleeding after surgery was likely to be more frequent in the fondaparinux group than in the enoxaparin group. The incidence of postoperative bleeding following colorectal surgery in previous studies ranged from 4.6 to 11.5% in patients with enoxaparin prophylaxis [17–20, 33] and 9.4 to 22.2% in patients with fondaparinux prophylaxis [16, 19, 21]. Our findings of major and minor bleeding in fondaparinux (10.4%) and enoxaparin (2.6%) prophylaxis are comparable to results from previous studies.

A recent study demonstrated the risk factors of postoperative bleeding in CRC patients administrated fondaparinux as thromboprophylaxis after surgery. They have shown that, in multivariate analysis, male gender, intraoperative blood loss of less than 25 mL, and a preoperative platelet count below 15 × 10^4^/μL were found to be independent risk factors for postoperative bleeding in the laparoscopic surgery group. Only the preoperative platelet count was an independent risk factor in the open surgery group [34]. We could not evaluate risk factors of postoperative bleeding differently between open and laparoscopic surgery due to the small number of patients in the present study. Moreover, they interestingly showed intraoperative blood loss as a negative impact for postoperative bleeding complication; however, we could not also find that intraoperative blood loss was significantly associated with negative risk for postoperative bleeding in univariate analysis.

The abnormalities of coagulation and fibrinolysis are frequently observed in cancer patients. A recent meta-analysis suggested that plasma D-dimer and fibrinogen levels were correlated with tumor stage and prognosis in digestive cancer patients [35–37]. High pretreatment plasma D-dimer was reported to predict poor survival of colorectal cancer [38, 39]. We also observed the elevation in preoperative and postoperative D-dimer levels as the increase in disease stage in our patients. Previous clinical studies have suggested that preoperative D-dimer may also predict the development of postoperative VTE [40, 41]. Therefore, advanced CRC patients with elevated levels of D-dimer have a high risk of VTE following colorectal surgery. On the contrary, we did not observe such hypercoagulation status in early disease-stage CRC patients in their clinical course. Our findings revealed that early disease-stage CRC was one of the risk factors for postoperative bleeding by univariate analysis. The hypercoagulation status accompanied with progression of CRC appeared to be related to postoperative bleeding following CRC surgery in patients with pharmacological thromboprophylaxis.

We acknowledge several limitations of the current study that was a retrospective, non-randomized, and small-number cohort study. First, generally, the number of events per variable (EPV) analyzed in logistic regression analysis is recommended as 10 or greater in the previous studies [14, 15]. The statistical power appeared to be relatively weak, since EPV in the present study (11 events per two variables) was lower than 10 in multiple logistic regression analysis. However, recent research has reported the possibility of relaxing the rule of 10 EPV in logistic and Cox regression analysis. Moreover, power calculation (1-*β* err prob) of multiple regression in this study was 0.8439 (effect size f2 0.05, *α* err prob 0.05, sample size 218, number of predictors 2), which was analyzed by G3*Power 3.1 [42]. Therefore, we believe that logistic regression analysis with 5-9 EPV in the present study was acceptable. Second, we found the significant difference in the incidence of postoperative bleeding between the two anticoagulants. We adapted enoxaparin as a pharmacological thromboprophylaxis for high-risk patients following CRC surgery in 2010. We switched enoxaparin to fondaparinux from June 2012 to April 2013, because enoxaparin must be administrated twice injection daily in contrast to once injection daily of fondaparinux in Japan. However, we often observed minor bleeding complications with the administration of fondaparinux. Therefore, we reinstated enoxaparin prophylaxis and found that the incidence of minor hemorrhagic complications with the enoxaparin was similar to the first term of enoxaparin administration. We could not continuously use fondaparinux for patients ethically after our finding the significant risk of bleeding events in patients administrated fondaparinux until the achievement in enough statistical power between two drugs. Although fondaparinux prophylaxis was identified as independent risk factors for postoperative bleeding in our study, additional prospective studies are needed to clarify the significant and true risk factors for bleeding complications between the two drugs.

## Conclusion

Fondaparinux administration and early disease-stage CRC appeared to be risk factors for postoperative bleeding in patients with pharmacological thromboprophylaxis undergoing surgical treatment for CRC. The coagulation status might be related to the occurrence of postoperative bleeding in patients with early disease-stage CRC according to perioperative D-dimer levels.

## Data Availability

The datasets used and analyzed during the current study are available from the corresponding author on reasonable request.

## Abbreviations

CRC: Colorectal cancer
IPC: Intermittent pneumatic compression
POD2: Second postoperative day
VTE: Venous thromboembolism
